# Superiority trial for the development of an ideal method for the closure of midline abdominal wall incisions to reduce the incidence of wound complications after elective gastroenterological surgery: study protocol for a randomized controlled trial

**DOI:** 10.1186/s13063-024-08167-w

**Published:** 2024-05-17

**Authors:** Shota Fukai, Yuki Mizusawa, Hiroshi Noda, Shingo Tsujinaka, Yukihisa Maeda, Ryuji Hasebe, Yusuke Eguchi, Rina Kanemitsu, Natsumi Matsuzawa, Iku Abe, Yuhei Endo, Taro Fukui, Yuji Takayama, Kosuke Ichida, Koetsu Inoue, Yuta Muto, Fumiaki Watanabe, Kazushige Futsuhara, Yasuyuki Miyakura, Toshiki Rikiyama

**Affiliations:** Department of Surgery, Saitama Medical Center, Jichi Medical University, 1-847 Amanuma-Cho, Omiya-Ku, Saitama, 330-8503 Japan

**Keywords:** Midline abdominal wall incision, Fascial closure, Peritoneal closure, Skin closure, Continuous suture, Interrupted suture, Incisional hernia, Surgical site infection, Wound complication

## Abstract

**Background:**

The recent guidelines from the European and American Hernia Societies recommend a continuous small-bite suturing technique with slowly absorbable sutures for fascial closure of midline abdominal wall incisions to reduce the incidence of wound complications, especially for incisional hernia. However, this is based on low-certainty evidence. We could not find any recommendations for skin closure. The wound closure technique is an important determinant of the risk of wound complications, and a comprehensive approach to prevent wound complications should be developed.

**Methods:**

We propose a single-institute, prospective, randomized, blinded-endpoint trial to assess the superiority of the combination of continuous suturing of the fascia without peritoneal closure and continuous suturing of the subcuticular tissue (study group) over that of interrupted suturing of the fascia together with the peritoneum and interrupted suturing of the subcuticular tissue (control group) for reducing the incidence of midline abdominal wall incision wound complications after elective gastroenterological surgery with a clean-contaminated wound. Permuted-block randomization with an allocation ratio of 1:1 and blocking will be used. We hypothesize that the study group will show a 50% reduction in the incidence of wound complications. The target number of cases is set at 284. The primary outcome is the incidence of wound complications, including incisional surgical site infection, hemorrhage, seroma, wound dehiscence within 30 days after surgery, and incisional hernia at approximately 1 year after surgery.

**Discussion:**

This trial will provide initial evidence on the ideal combination of fascial and skin closure for midline abdominal wall incision to reduce the incidence of overall postoperative wound complications after gastroenterological surgery with a clean-contaminated wound. This trial is expected to generate high-quality evidence that supports the current guidelines for the closure of abdominal wall incisions from the European and American Hernia Societies and to contribute to their next updates.

**Trial registration:**

UMIN-CTR UMIN000048442. Registered on 1 August 2022. https://center6.umin.ac.jp/cgi-open-bin/ctr_e/ctr_view.cgi?recptno=R000055205

## Introduction

### Background and rationale {6a, 6b}

Wound complications are among the most common morbidities for patients after gastroenterological surgery [1–4]. Short-term postoperative wound complications include incisional surgical site infection (SSI) [1, 2], hematoma, seroma, and wound dehiscence. Incisional hernia (IH) accounts for the majority of long-term postoperative wound complications [3, 4]. Both short-term and long-term wound complications are associated with a decreased quality of life, increased risks of morbidity and mortality, and dramatically increased medical costs [1–5]. Obesity, smoking, and old age are well-known risk factors for IH and highly overlap with the risk factors for SSI and wound dehiscence. Furthermore, SSI itself is an important risk factor for IH, and wound dehiscence may predict IH [6]. Thus, the pathogenesis and development of all wound complications may be correlated with each other, and a comprehensive approach to reduce the overall incidence of wound complications should be developed.

The wound closure technique that is used to close an abdominal wall incision is an important determinant of the risk of developing wound complications, especially in IH [3, 4]. The European Hernia Society proposed guidelines on the closure of abdominal wall incisions in 2015 [3], and it was recently updated by the European and American Society in 2022 [4]. For closure of the fascia, they recommend the combined use of a continuous small-bite suturing technique with slow absorbable sutures to reduce the risk of IH [3, 4].

Although the guidelines recommend the continuous suture technique, there has been no high-quality evidence suggesting the superiority of the continuous suture technique to the intermittent suture technique for the prevention of wound complications. Reliable recent meta-analyses that compared the interrupted and continuous suture techniques showed that there was no difference in the incidence of HH, wound dehiscence, or SSI [7, 8]. These meta-analyses included many smaller and older studies of low-certainty evidence; therefore, the evidence is of poor quality [7, 8]. Accordingly, for the establishment of high-quality evidence of wound closure techniques, further studies should be conducted to compare the impact of interrupted and continuous suture techniques in the prevention of wound complications.

For the closure of a midline abdominal wall incision (MAWI) in elective surgery, single-layer aponeurotic closure is recommended, but not mass closure in an all-in-one approach that includes the fascia, muscle, and peritoneum [3]. In a systematic review, there was no evidence of any short-term or long-term advantages of peritoneal closure during the closure of the laparotomy incision [9], and it was not recommended in the guidelines [3]. However, there was no evidence that mass versus layered closure increased the risk of IH, and no clinical studies directly comparing mass closure and a single-layer aponeurotic closure were found [3]. Thus, the recent recommendation appears to be based on low-certainty evidence, and further research is required to make strong recommendations [3, 4].

For skin closure, we could not find any recommendation for the prevention of wound complications. Subcuticular sutures for skin closure have been revealed to be associated with a lower incidence of wound complications and better cosmesis in clean wounds [10, 11]. In clean-contaminated wounds after gastroenterological surgery, subcuticular sutures were associated with a lower incidence of incisional SSI in comparison to staples in sub-analyses in some trials, but the effect of subcuticular sutures for preventing wound complications, including incisional SSI, has been controversial [12, 13]. A systematic review suggested that the use of continuous subcuticular sutures may reduce the risk of superficial wound dehiscence in comparison to interrupted sutures; however, there is uncertainty because of the quality of the evidence [14].

In gastroenterological surgery, MAWIs are widely used because they enable access to the whole abdominal cavity in open surgery. With the development of surgical technology since the 1990s, surgical procedures have progressed from open surgery to minimally invasive surgery, such as laparoscopic surgery and robotic surgery. With current surgical practices in developed countries, the proportion of minimally invasive surgery among gastroenterological surgeries is reported to be approximately 70% [15, 16]. Minimally invasive surgery has been revealed to reduce IH as an SSI [4]. However, the use of an MAWI as a specimen extraction site was found to be associated with a higher incidence of IH in comparison to nonmidline incision sites [4]. An MAWI is most frequently used as a site for specimen extraction in minimally invasive surgery because it achieves better cosmesis; thus, the development of an ideal method for MAWI closure after gastroenterological surgery is necessary for both open and minimally invasive surgery.

### Objective {7}

This single-center, prospective, randomized controlled trial (RCT) is being performed to evaluate the superiority of the combination of MAWI closure with continuous suturing of the fascia using a single-layer aponeurotic technique and continuous suturing of the subcuticular tissue for skin closure. The purpose of this trial is to establish the ideal combination of fascial closure and skin closure for reducing the overall incidence of MAWI wound complications after gastroenterological surgery.

### Study design {5a, 8}

This is a single-institute, prospective, randomized, blinded-endpoint trial being conducted to evaluate the superiority of the combination of continuous suturing of the fascia without peritoneal closure (single-layer aponeurotic closure) and continuous suturing of the subcuticular tissue over that of interrupted suturing of the fascia, together with the peritoneum and interrupted suturing of the subcuticular tissue, for reducing the incidence of MAWI wound complications after elective gastroenterological surgery with a clean-contaminated wound. This trial was designed and is being conducted by Saitama Medical Center, Jichi Medical University, and the present protocol follows the recommendations outlined in the Standard Protocol Items: Recommendations for Interventional Trials guidelines for RCTs (Supplementary Material 1) [17]. This trial was registered in the UMIN-CTR, and the WHO Trial Registration Data Set is included and can be found within the registry.

## Methods: participants, intervention, and outcomes

### Study setting {9}

Patients who receive elective gastroenterological surgery in the Department of Surgery, Saitama Medical Center, Jichi Medical University, and who are able to understand the extent and nature of this trial are eligible for inclusion in this study. This trial was approved by the Bioethics Committee for Clinical Research, Saitama Medical Center, Jichi Medical University (S22-001).

### Eligibility criteria {10}

The inclusion criteria are as follows:

- 1.Scheduled to undergo elective surgery for the esophagus, stomach, duodenum, jejunum, ileum, colorectal, pancreas, liver, or biliary tract with a class II (clean-contaminated) surgical wound (Table 1)
- 2. Age ≥ 18 years at the time of consent being obtained by nonblinded investigators.
- 3. Scheduled to undergo computed tomography 1 year after surgery.
- 4. Written informed consent provided.

**Table 1:** Definition of the wound classes

Class I (clean)	An uninfected operation wound in which no inflammation is encountered and the respiratory, alimentary, and genitourinary tract is not entered
Class II (clean-contaminated)	An operative wound in which the respiratory, alimentary, and genitourinary tracts are entered under controlled conditions and without unusual contamination provided no evidence of infection or major break in technique is encountered
Class III (contaminated)	A wound in which gross contamination/spillage and a break in sterile technique occurs, and an incision in which acute, nonpurulent inflammation is encountered
Class IV (dirty-contaminated)	A wound that is already considered infected, such as old traumatic wounds with retained devitalized tissue or perforated viscera

The exclusion criteria are as follows:

- 1. Emergency surgery.
- 2. Age < 18 years.
- 3. A history of midline abdominal wall incision.
- 4. A history of abdominal incisional hernia or fascial tear.
- 5. Identification of bacterial infection in the surgical field or the use of antibiotic therapy prior to the operation.
- 6. Presence of a contaminated abdominal cavity due to stoma, intestinal fistula, or drainage tube.
- 7. Open wound management for prior operation.
- 8. Synchronous operation for more than two targeted organs.
- 9. Current immunotherapy (≥ 40 mg of a corticosteroid per day or azathioprine).
- 10. Chemotherapy within 14 days prior to surgery.
- 11. A history of abdominal radiotherapy.
- 12. Pregnancy.
- 13. Registered for another clinical trial.
- 14. Conditions that make the patient unsuitable for inclusion according to the judgment of nonblinded investigators.

### Who will take informed consent? {26a}

Nonblinded investigators who received ethics education and were approved by the Bioethics Committee for Clinical Research, Saitama Medical Center, Jichi Medical University, will obtain informed consent for surgery and inclusion in the clinical trial after admission, 1–2 days before surgery. Adequate time will be given to participants to consider their decision regarding trial participation. Subsequently, participants can sign the informed consent form, and they can withdraw at any time during the trial.

### Additional consent provisions for collection and use of participant data and biological specimens {26b}

Patient characteristics, such as sex, age, body mass index, serum albumin level, comorbidities, American Society of Anesthesiologists-physical status classification and preoperative treatment, will be collected. In addition, surgical data, such as the surgical procedure, operative time, estimated blood loss, wound classification, and length of postoperative hospital stay, will be collected. Additional biological specimens will not be collected for this trial. The investigators do not expect to conduct ancillary studies requiring the use of participant data that is collected in this study.

### Interventions

#### Intervention description {11a}

Study group:MAWI closure with continuous suture of the fascia without peritoneum (single layer aponeurotic technique) and continuous suture of the subcuticular tissue for skin closure.

Control group:MAWI closure with interrupted suture of the fascia with the peritoneum and interrupted suture of the subcuticular tissue for skin closure.

In the study group, the fascia is sutured continuously with 1-STRATAFIX SYMMETRIC PDS plus (Ethicon, Johnson & Johnson, Somerville, New Jersey, USA) without the peritoneum. Continuous subcuticular suture is then used for skin closure using 4–0 STRATAFIX Spiral PDS plus (Ethicon, Johnson & Johnson, Somerville, New Jersey, USA). In the control group, the peritoneum and fascia are sutured together using interrupted sutures with 1-PDS plus (Ethicon, Johnson & Johnson, Somerville, New Jersey, USA). Interrupted subcuticular sutures are then used for skin closure using 4–0 PDS plus (Ethicon, Johnson & Johnson, Somerville, NJ, USA). In both groups, narrow and closed fascia sutures, where each suture will be placed approximately 5–9 mm from the edge of the fascia and approximately 5 mm from the adjacent fascia suture, are recommended. Suturing of the fascia and subcuticular tissue is applied from both ends of the incision toward the center or applied from one end to the other end according to the operator’s direction during the operation.

#### Criteria for discontinuing or modifying allocated interventions {11b}

There are no predominant criteria for discontinuing or modifying the intervention assigned to participants. All individuals are participating on a voluntary basis, and they have the option to withdraw from the study at any time for any reason without facing any negative consequences.

#### Strategies to improve adherence to interventions {11c}

All participants who meet the criteria will receive a participant information sheet from the investigators before giving their written informed consent. By enrolling participants who were scheduled to undergo computed tomography 1 year after surgery, we ensured that adherence to the study protocol would improve. Furthermore, participants will be informed of the significance of completing follow-up assessments.

#### Relevant concomitant care permitted or prohibited during the trial {11d}

The following measures are used to prevent SSI in our protocol:

- 1. Surgical skin antisepsis with aqueous 10% povidone–iodine solution is performed before skin incision.
- 2. Standard antibiotic prophylaxis is administered 30 min before making the skin incision with additional doses every 3 h for patients with a normal renal function.
- 3. The use of a wound protector is recommended.
- 4. Surgical gloves are changed before skin suture.
- 5. Intraoperatively and postoperatively, a normal body temperature is maintained using warming devices and appropriate oxygenation.
- 6. Intraoperative wound irrigation was performed for 1 min with 40 mL of aqueous 10% povidone–iodine (POVIDONE–IODINE solution 10% “MEIJI”; Meiji Seika Pharma Co, Ltd, Tokyo, Japan).
- 7. Perioperative glycemic control is implemented with a blood glucose target level of < 200 mg/dL.

All suture materials were antimicrobial-coated slowly absorbable monofilaments. Before the initiation of and during the trial, investigator meetings were held several times, and the treatment protocol was fully disseminated to all investigators. Investigators who were not familiar with continuous suture techniques were trained in the required skills using pig abdominal wall models and performed operations under the direction of surgeons who were familiar with the technique before participating in the trial. The principal investigator is responsible for all trial-related issues and questions.

All participants will receive standard postoperative care management during hospitalization and throughout the duration of the study.

#### Provisions for posttrial care {30}

There are no provisions for participants who participate in the trial. Posttrial care follows the standards of care after surgery.

### Outcomes {12, 18a}

#### Primary outcome

The primary outcome is the incidence of wound complications, including incisional SSI, hemorrhage, seroma, and wound dehiscence within 30 days after surgery and IH at approximately 1 year after surgery. Nonblinded investigators will check the surgical wound and describe the medical records during hospitalization. If wound complications are suspected based on the clinical findings, nonblinded investigators will record the treatment details in the medical record. After discharge, participants will be referred to the outpatient department approximately 30 days after surgery. Participants will be recommended to contact us and visit the outpatient department soon if they experience any symptoms suggesting wound complications. Nonblinded investigators will examine the patients in the same way as during hospitalization. One year after surgery, participants will be referred to the outpatient department, and the incidence of incisional hernia will be assessed with a physical examination by nonblinded investigators and computed tomography (CT) of the abdomen. The blinded assessors will determine the presence or absence of wound complications according to the clinical records.

Incisional SSI is defined according to the standard criteria devised by the CDC [18]. Incisional SSI includes superficial and deep incisional SSI that develops during the first 30 days after surgery. Superficial incisional SSI involves the skin or subcutaneous tissue at the incision site, and deep incisional SSI affects more internal structures of the abdominal layer (such as the fascia or muscle). Wound hemorrhage is defined as superficial postoperative wound bleeding or subcutaneous hematoma. Seroma is defined as a collection of serous fluid in the subcutaneous space, detected either clinically or by imaging. Wound dehiscence includes superficial dehiscence and total dehiscence. Superficial dehiscence is defined as skin-only dehiscence. Total dehiscence is defined as a dehiscence of the entire abdominal wall. IH is defined as any abdominal wall gap with or without a bulge in the area of a postoperative scar that is perceptible or palpable by clinical examination or imaging according to the European Hernia Society guidelines [19].

#### Secondary outcomes

The secondary outcomes are the duration of abdominal wall closure and wound pain on postoperative day (POD) 7. Nonblinded operating surgeons and nurses will measure and record the duration of abdominal wall closure, wound length after the completion of wound closure, and the number of operating surgeons involved in wound closure. The duration of wound closure is defined as the time from the application of the first suture needle to the wound until the suture is finished and the thread is cut. A questionnaire using a 10-point numerical rating scale is used for the estimation of wound pain [20]. It is documented by the participant on POD7 and is collected by blinded investigators.

### Participant timeline {13}

The participant timeline is shown in Table 2. Nonblinded investigators will obtain informed consent for surgery and inclusion in the clinical trial after admission 1–2 days before surgery. We will confirm and record each patient’s medical history, allergies, and physical examination results. After their written informed consent has been obtained, patients eligible for this trial will be randomized into two groups before surgery. The observation period will be approximately 1 year after surgery. The 1-year follow-up visit is defined as a follow-up visit up to 15 months after surgery. Table 2 shows a summary of the schedule and the data collected for this trial.

**Table 2:** Schedule and data collection of this trial

	Study period						
	Before allocation	After allocation			Follow-up		
**Timepoint**	1–2 days	Surgery	POD1	POD3	POD4–29	POD30	9–15 months
**Screening**	X						
Eligibility screen	X						
Informed consent	X						
Randomization		X					
**Interventions**		X					
Intervention A		X					
Intervention B		X					
**Assessments**							
Demographic data	X						
Medical history	X						
Physical examination	X						
Blood sample	X		X	X			
Type of operation		X					
Time of operation		X					
Wound classification		X					
Estimated blood loss		X					
Blood transfusion		X					
Stoma creation		X					
Documentation of wound complications			X	X	X	X	
Wound swab microbiology			X	X	X	X	
Documentation of reoperation			X	X	X	X	
Documentation of AE			X	X	X	X	
Duration of hospital stay						X	
Computed tomography							X
Documentation of incisional hernia							X

*AE*, adverse effect

### Sample size {14}

**Fig. 1:**
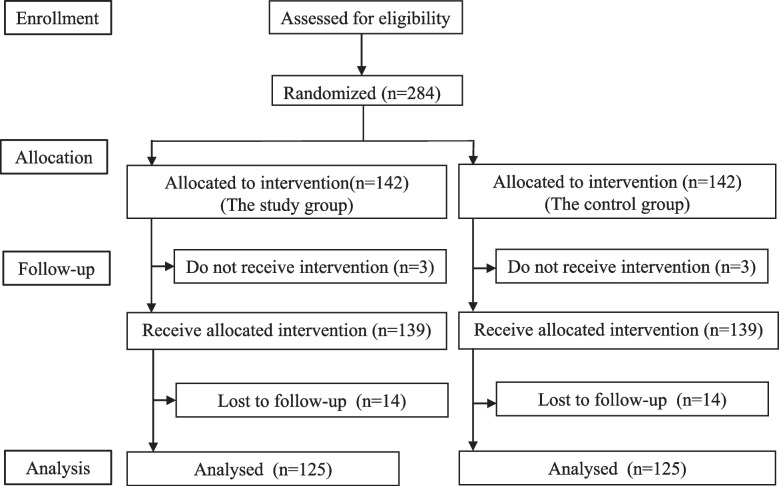
CONSORT flow chart

A retrospective cohort of patients who underwent gastrointestinal surgery and MAWI closure (in which the peritoneum and fascia were sutured together using interrupted sutures with 1-PDS plus, with subcutaneous interrupted sutures then used for skin closure using 4–0 PDS) at our department from June 2019 to December 2020 was analyzed. The incidence rates of each complication were as follows: incisional SSI (7.6%), hematoma (5.1%), 5.9% seroma (5.9%), superficial dehiscence (1.4%), total dehiscence (0%), and IH at approximately 1 year after surgery (16.8%). When a patient developed more than 2 wound complications, the number of patients who developed wound complications was assumed to be one. In total, the incidence of wound complications was 29.2%. In this trial, we hypothesized that midline abdominal wall incision closure with continuous suturing of the fascia using a single-layer aponeurotic technique and continuous suturing of the subcuticular tissue would achieve a 50% reduction in the incidence of wound complications. The expected wound complication rates of the study and control groups were 14.6% and 29.2%, respectively. Power and Sample Size Calculation version 3.1.6 was used for sample size estimation [21]. With a two-sided alpha level of 0.05, it is estimated that a total of 250 patients will be needed for the trial to have 80% power to detect superiority in the reduction of the frequency of wound complications. Two percent of cases are expected to be excluded after allocation, and 10% of cases are expected to drop out during the postoperative follow-up period. Thus, the total target number of cases is set at 284 (Fig. 1).

### Recruitment {15}

This trial was approved by our institutional review board on June 30, 2022, and is registered in the University Hospital Medical Information Network Clinical Trial Registry (part of the WHO International Clinical Trial Registry Platform) under the identification number UMIN-CTR000048442. Patients who receive elective gastroenterological surgery will be considered candidates for trial enrollment in concordance with the inclusion and exclusion criteria. Informed consent will be obtained from all participants by investigators. We are confident that we can enroll a sufficient number of eligible patients, as our institution conducts over 300 elective gastroenterological surgeries with clean-contaminated wounds annually.

## Assignment of interventions: allocation

### Sequence generation {16a}

Participants will be registered, randomized, and allocated by nonblinded investigators. Participants’ data will be password-protected and will only be accessible by investigators. All access to the secure separate database will be monitored and logged. Permuted-block randomization with an allocation ratio of 1:1 and blocking will be used. The randomization will be stratified by sex, surgical organ (upper gastrointestine, small bowel, colorectum, hepatobiliary–pancreas, and others), and surgical approach (laparotomy or laparoscopy).

### Concealment mechanism {16b}

A web-based block randomization system (Research Electronic Data Capture [REDCap]) will be used to generate the randomization sequence [22]. Both the randomization methodology and the allocation sequence will be concealed from both the assessor and the participants.

### Implementation {16c}

An independent investigator will generate a stratified randomization list for the allocation sequence. Investigators will enroll and assign participants to interventions.

## Assignment of interventions: blinding

### Who will be blinded {17a}

Patients will be blinded to their assigned group. However, the operating surgeons cannot be blinded. The assessors will be blinded, as they will not be in the operating room and cannot access the randomization results. The data on wound complications and analyses will be entered by blinded investigators. The biostatistician will be blinded.

### Procedure for unblinding if needed {17b}

As investigators are aware of the participant allocation, unblinding will not be needed.

## Data collection and management

### Plans to promote participant retention and complete follow-up {18b}

Participants are patients who require regular follow-up and are scheduled to undergo computed tomography 1 year after surgery. We expect that participants will be available for follow-up due to concerns about disease recurrence.

### Data management {19}

The study will be conducted according to good clinical practice standards and legal regulations. Prior to inclusion, patients will be informed that any patient-related data and materials will be appropriately pseudonymized and that these data may be used for analytical and publication purposes. All information required by the study protocol and collected during this trial will be entered in the electronic case report form (CRF; encrypted Excel database) by investigators. The data entry module contains an online range and logical checks. All data will be collected by the investigators in an anonymous and encrypted database. The confidentiality of the participants will be maintained at all times. The investigator will maintain all study-related information, including medical records, CRFs, written informed consent documents, and other pertinent data, for 5 years after trial termination.

### Confidentiality {27}

The investigators will obtain the participants’ information from medical records and collect the information in a password-protected file in the hospital database. The participants’ hospital identification will be anonymized. Datasets will only include summary data, which cannot identify individual participants. These will be presented in the manuscript.

### Plans for collection, laboratory evaluation, and storage of biological specimens for genetic or molecular analysis in this trial/future use {33}

Not applicable as no biological specimens will be collected in this study.

## Statistical methods

### Statistical methods for primary and secondary outcomes {20a}

All analyses will be performed after the termination of the main part of the trial, that is, after the last 1-year follow-up visit has taken place. We will perform the primary analyses using the full analysis set, from which patients who do not undergo surgery or who withdraw their consent before the assessment of the primary endpoint are excluded (modified intention-to-treat set). In addition, we will repeat the analyses in the per-protocol set, further excluding patients with major protocol deviations. Student’s *t* test or the Mann–Whitney *U* test will be used to compare continuous variables with a normal or nonnormal distribution. The *χ*^2^ test or Fisher’s exact test will be used to compare categorical variables between the study group and the control group. *P* values of < 0.05 were considered to indicate statistical significance. All statistical analyses will be conducted using EZR [23].

### Interim analyses {21b}

We have no plans to conduct any interim analyses since both interventions are associated with a low degree of risk.

### Method for additional analyses (e.g., subgroup analyses) {20b}

Subgroup analyses will be performed for the surgical approach (laparotomy or laparoscopy). *P* values of < 0.05 will be considered statistically significant.

### Methods in the analysis to handle protocol nonadherence and any statistical methods to handle missing data {20c}

If missing data for any variable exceed 5%, multiple imputations will be used to handle missing values.

### Plans to give access to the full protocol, participant-level data, and statistical code {31c}

The collection of data underlying this article is in progress. When data collection and follow-up are finalized, data from the study will be available on reasonable request from the corresponding author.

## Oversight and monitoring

### Composition of the coordinating center and trial steering committee {5d}

The authors will coordinate and steer this study.

### Composition of the data monitoring committee, its role, and reporting structure {21a}

We do not have the composition of the data monitoring committee. During the study period, data monitoring and surgical monitoring for this trial will be conducted by blinded investigators who are not participating in this trial.

### Adverse event reporting and harms {22}

Adverse effects can be part of the outcome measures detailed earlier in the protocol or other unspecified side effects. MAWI closure with continuous or intermittent suturing of the fascia and subcutaneous tissue is generally performed in daily surgical practice. STRATAFIX SYMMETRIC PDS plus, 4–0 STRATAFIX Spiral PDS plus, PDS plus, and 4–0 PDS plus (Ethicon, Johnson & Johnson, Somerville, New Jersey, USA) are used in a manner that is in line with the Pharmaceutical Affairs Law.

Adverse events cover any signs, symptoms, syndrome, or illness that appears or worsens in a patient during the observation period in the clinical trial and that may impair the wellbeing of the patient. Any adverse events that require additional pharmacological, interventional, or surgical management within the study periods will be collected by non-blinded investigators and monitored by blinded investigators who are not participating in this trial. Adverse events will be regarded as postoperative complications and graded according to the Clavien-Dindo classification [24]. A serious adverse event is any adverse event that occurs at any time during the observation period that results in death, is immediately life-threatening, requires or prolongs hospitalization, results in persistent or significant disability or incapacity, or causes congenital anomalies in offspring. Each participant will receive informed consent about notification and follow-up of adverse events and will be provided with medical care for any potential harm stemming from their participation in the trial. Documentation and relevant patient data on all serious adverse events will be submitted to the Bioethics Committee for Clinical Research, Saitama Medical Center, Jichi Medical University, and the committee will decide on the final diagnostic classification of critical clinical events and make recommendations on whether this trial should be stopped or not.

### Frequency and plans for auditing trial conduct {23}

The progress of the trial will be updated on the web page of UMIN-CTR every 6 months, and the Bioethics Committee for Clinical Research, Saitama Medical Center, Jichi Medical University will monitor progress approximately every year. All data will be collected by the investigators in an anonymous and encrypted database. The confidentiality of the participants will be maintained at all times. The investigator will maintain all study-related information, including medical records, CRFs, written informed consent documents, and other pertinent data, for 5 years after trial termination.

### Plans for communicating important protocol amendments to relevant parties (e.g., trial participants, ethical committees) {25}

If the protocol needs to be modified, it will be reviewed again by the ethics committee, and upon approval, the trial registry and protocol will be updated.

### Dissemination policy {31a, 31b}

The final results will be reported in international peer-reviewed journals immediately after the completion of the trial. All authorships will be in accordance with the International Committee of Medical Journal Editors guidelines. We do not intend to use professional writers.

## Discussion

This study is designed to establish the ideal combination of fascial and skin closure of a clean-contaminated MAWI wound after gastroenterological surgery for reducing the incidence of overall wound complications. No previous studies have evaluated the ideal combination of fascial and skin closure of MAWI.

High-quality studies adhere to methodological principles to minimize errors in surgical trials, including adequate randomization, concealment of allocation, blinding, performance of an intention-to-treat analysis, complete follow-up, reliable accurate outcome measures, and an a priori sample size calculation [25]. The present trial is designed according to these principles. Our inclusion and exclusion criteria for participants aim to select homogenous patients with clean-contaminated wounds. The primary outcome of this trial is the incidence of wound complications, including subcutaneous hematoma, seroma, incisional SSI, wound dehiscence, and IH. Incisional SSI is defined according to the standard criteria devised by the CDC, which have been used for research, quality improvement, public reporting, and pay-for-performance comparisons; the definition is considered the most reliable [18]. IH is defined according to the European Hernia Society guidelines, either clinically or by imaging [19]. CT is reliable and reproducible, and therefore, the inclusion criteria demand that patients are scheduled to undergo CT 1 year after surgery [3, 4, 19]. Thus, reliable accurate outcome measures are used. In the retrospective cohort of patients used for the calculation of the target number of this trial, SSI and IH are also judged using these definitions. The incidence rates of hemorrhage or hematoma, incisional SSI, seroma, wound superficial or complete dehiscence, and IH in a retrospective cohort are almost equal to the general incidence rates [1–4, 7–14]. There have been no studies evaluating the ideal combination of fascial and skin closure of MAWIs for reducing the incidence of wound complications. Therefore, we hypothesized that MAWI closure with continuous suturing of the fascia using a single-layer aponeurotic technique and continuous suturing of the subcuticular tissue achieves a 50% reduction in the incidence of wound complications because it is clinically meaningful in patients undergoing elective gastroenterological operations. In addition, the number of dropout cases was predicted based on two previous RCTs conducted by our department [16, 26, 27] and the protocols of previous RCTs for MAWI closure [28–30]. Thus, the total number of cases might be as accurate as possible. As 50–70% of IHs occur within 1 year after the operation, we decided that the postoperative follow-up period would be 1 year, and the incidence of IH at this time was the primary end-point of this trial [28–30]. Because this RCT was conducted in a single center, the follow-up of outcomes can be expected to be more complete in comparison to multicenter RCTs.

In this trial, multiple perioperative measures for SSI prevention are implemented according to clinical guidelines [31, 32]. The implementation of a set of evidence-based practices for the prevention of SSI has been proven to reduce the incidence of incisional SSI in gastroenterological surgery [33]. Recent studies have shown that the small bite technique for MAWI closure is effective for the prevention of IH. These studies were of acceptable quality; thus, the technique has been incorporated into the updated guidelines [4, 34–36]. In addition, the implementation of the small bite technique for MAWI closure in clinical practice may be correlated with a reduction in incisional SSI [37, 38]. Irrespective of the applied suture technique, slowly absorbable sutures were revealed to be associated with a significantly lower incidence of IH in the INLINE study [39]. Therefore, the small bite technique using slowly absorbable sutures is implemented in both groups. The efforts in this trial might determine the precise incidence of wound complications under appropriate management based on the current guidelines.

The present study is associated with some limitations. First, it includes all patients undergoing gastroenterological surgery, regardless of the organ, diagnosis, or procedure. Second, the trial is conducted in a single center, and single-center RCTs typically show greater treatment effects than multicenter RCTs [25]. A well-designed multicenter RCT will be needed to generalize and demonstrate the results of this study.

In conclusion, this trial will provide initial evidence on the ideal combination of fascial and skin closure for MAWI to reduce the incidence of overall postoperative wound complications after gastroenterological surgery with a clean-contaminated wound. This trial is expected to generate high-quality evidence that supports the current guidelines for the closure of abdominal wall incisions from the European and American Hernia Societies and to contribute to their next updates.

### Protocol version and trial status {3}

Recruitment is continuing steadily, and as of December 25, 2023, 154 participants have been enrolled. The current protocol is in operation at version 1.2 (January 4, 2023). Recruitment is expected to end in December 2024.

## Data Availability

Deidentified participant data that underlie the results reported in this article will be made available to researchers who provide a methodologically sound proposal. Proposals should be directed to Hiroshi Noda (noda164@omiya.jichi.ac.jp); to gain access, data requestors will need to sign a data access agreement.
